# Combined analysis of finite element model and audiometry provides insights into the pathogenesis of conductive hearing loss

**DOI:** 10.3389/fbioe.2022.967475

**Published:** 2022-09-02

**Authors:** Motoki Hirabayashi, Sho Kurihara, Ryuya Ito, Yuta Kurashina, Masaomi Motegi, Hirotaka James Okano, Yutaka Yamamoto, Hiromi Kojima, Takumi Asakura

**Affiliations:** ^1^ Department of Otorhinolaryngology, The Jikei University School of Medicine, Tokyo, Japan; ^2^ Division of Regenerative Medicine, The Jikei University School of Medicine, Tokyo, Japan; ^3^ Department of Mechanical Engineering, Faculty of Science and Technology, Tokyo University of Science, Tokyo, Japan; ^4^ Department of Mechanical Systems Engineering, Faculty of Engineering, Tokyo University of Agriculture and Technology, Tokyo, Japan

**Keywords:** vibroacoustic analysis, finite element model, middle ear impedance, audiogram, conductive hearing loss, air-bone gap, ossicular chain disruption, otosclerosis

## Abstract

The middle ear transmits sound to the inner ear *via* vibrations in the eardrum and ossicles, and damage to the middle ear results in conductive hearing loss. Although conductive hearing loss can be corrected by surgery, the currently available clinical investigations cannot always diagnose the ossicular chain pathology underlying the conductive hearing loss, and even intraoperative findings can be equivocal. Acoustic analysis using finite element models (FEMs) can simulate the sound pressure change at an arbitrary site for each frequency. FEMs are used in acoustic engineering to simulate the frequency-dependent sound pressure distribution at discrete cells in a simulated model and analyze the effects of specific parameters on the audiogram. However, few reports have compared the numerical results obtained using FEMs with data from clinical cases. We used FEMs to simulate audiograms of the air-bone gap (ABG) for various ossicular chain defects and compared these with preoperative audiograms obtained from 44 patients with a normal tympanic membrane who had otosclerosis, middle ear malformations or traumatic ossicular disruption. The simulated audiograms for otosclerosis and attic fixation of the malleus-incus complex both exhibited an up-slope but could be distinguished from each other based on the ABG at 1000 Hz. The simulated audiogram for incudostapedial joint discontinuity exhibited a peak at around 750 Hz and a down-slope above 1000 Hz. In general, the simulated audiograms for otosclerosis, attic fixation and incudostapedial joint discontinuity were consistent with those obtained from clinical cases. Additional simulations indicated that changes in ossicular mass had relatively small effects on ABG. Furthermore, analyses of combination pathologies suggested that the effects of one defect on ABG were added to those of the other defect. These FEM-based findings provide insights into the pathogenesis of conductive hearing loss due to otosclerosis, middle ear malformations and traumatic injury.

## Introduction

The mammalian middle ear contains three bones (malleus, incus and stapes) suspended by ligaments and tendons. The middle ear acts as a “bridge” that transmits sound vibrations between the tympanic membrane and oval window. Among patients with an intact tympanic membrane and an air-filled middle ear cavity, the main causes of conductive hearing loss with a large air-bone gap (ABG) in the audiogram are abnormalities of the ossicular chain (fixation or discontinuity) and the presence of a third window in the inner ear (Merchant and Rosowski, 2010).

Disorders that cause ossicular chain abnormalities include congenital malformations, otosclerosis, trauma and inflammatory diseases. During normal development, the three ossicles separate from the temporal bone and become linked (Teunissen and Cremers, 1993; Bartel-Friedrich and Wulke, 2007; Yamamoto et al., 2014). Malformations are most frequently due to loss of the long process of the incus, sclerosis of the footplate of stapes, or fixation of the malleus-incus complex to the attic wall of the middle ear. Otosclerosis is a condition caused by abnormal bone remodeling around the stapes that results in progressive hardening of the annular ligament of the footplate. Traumatic injuries include dislocation and separation of the incudomalleolar or incudostapedial joints between the ossicles (Meriot et al., 1997) and ossicular fractures, which most commonly occur in the long process of the incus (Hough and Stuart, 1968; Wright et al., 1969). Since more than 80% of cases of conductive hearing loss are due to otitis media with cholesteatoma or chronic suppurative otitis media, the diagnosis of congenital malformation or otosclerosis is often delayed. Delays in the diagnosis of traumatic injury also occur due to the high incidence of indirect injury (Delrue et al., 2016). However, it is extremely important to diagnose ossicular chain defects and understand their pathogenesis because these defects can be surgically corrected.

Conductive hearing loss can be treated by surgical reconstruction, but the operative difficulty depends on the location and severity of the defect and the presence of concomitant pathology. Inadequate knowledge of the underlying pathology can lead to the lesion being missed and the operation taking longer than expected. In fact, even detailed intraoperative microscopy cannot fully visualize the surgical field if the view is obstructed by cholesteatoma or the fracture line is in close proximity to soft tissue (Farahmand et al., 2016; Sarmento et al., 2017). Therefore, it is essential that the underlying cause of conductive hearing loss is understood before surgery.

Preoperative investigations include otoscopy, computed tomography (CT) and audiometry. Otoscopy is the most basic examination used to assess the condition of the middle ear and can detect middle ear effusion, negative pressure in the middle ear and increased mobility of the tympanic membrane. However, otoscopy provides only limited information about the continuity and vibratory properties of the ossicles because only the handle of the malleus is visible. The resolution of CT scanning has improved in recent years, which has enabled the ossicles to be visualized more clearly with this technique. However, ossicular fixation and complete or incomplete discontinuity of the ossicular chain are difficult to detect using CT. Audiological tests such as pure-tone audiometry and tympanometry can quantify the efficiency of sound transmission and the impedance in the middle ear, and the results of these tests are known to reflect middle ear pathology. In particular, pure-tone audiometry contains frequency-specific information that can be used to estimate microscopic and qualitative changes in pathology.

There have been many attempts to infer pathology from the results of pure-tone audiometry, and this has contributed to clinical practice. For example, an up-sloping audiogram due to an elevated low-frequency threshold is typical of otosclerosis, and large increases in threshold at all frequencies are seen in patients with ossicular chain dislocation. However, the relationships between audiometry findings and underlying diseases have been inferred from clinical data, and the specific effects of each type of pathology on sound transmission efficiency have yet to be fully characterized. Therefore, preoperative pure-tone audiometry has limited ability to characterize the specific pathology affecting the ossicular chain.

In view of the diagnostic limitations described above, we have focused on the use of finite element analysis (FEA) to evaluate the middle ear sound transmission system. Phantom simulations allow the visualization of nanoscale vibrations in arbitrary structures within the simulated middle ear, and the physical properties of these structures can be altered at will. Finite element models (FEMs) have improved over time and been applied not only to the middle ear but also to the outer ear and inner ear. The first FEM of the middle ear was published by Funnell and Laszlo in 1978, and their model of the tympanic membrane of the cat was shown to be consistent with experimental values (Funnell and Laszlo, 1978). Since then, many groups have used FEMs to study the dynamics of the tympanic membrane and ear ossicles in cats (Ladak and Funnell, 1996), gerbils (Elkhouri et al., 2006; Aernouts and Dirckx, 2011), rabbits (Aernouts et al., 2010) and humans (Wada et al., 1992; Beer et al., 1999; Koike et al., 2002). A complete FEM of the human middle ear was published by Gan et al., in 2004, and a model of the cochlea was added in 2007 (Gan et al., 2004; Gan et al., 2007).

The aim of the present study was to construct FEMs that could simulate the pathogenesis of conductive hearing loss due to otosclerosis, middle ear malformation or trauma. We compared the results of our FEMs with data obtained from patients who had undergone surgery for hearing loss and whose pathology was known from the intraoperative findings. First, we evaluated the validity of a newly constructed FEM of a normal middle ear by comparing the results with previous reports. Then, we constructed models of ossicular sclerosis and incomplete ossicular discontinuity and compared the findings with audiograms obtained from real cases. Finally, we used FEMs to analyze the effects of changes in ossicular chain mass and complex pathologies. The results of our FEA show that, for each of the diseases that cause conductive hearing loss, the simulated audiometric pattern was generally consistent with the audiograms of actual cases. Based on the findings of this study, we suggest that the combination of pure-tone audiometry and FEA could be used to improve the preoperative diagnosis of the pathology underlying conductive hearing loss.

## Materials and methods

### Construction of the finite element model

**FIGURE 1:**
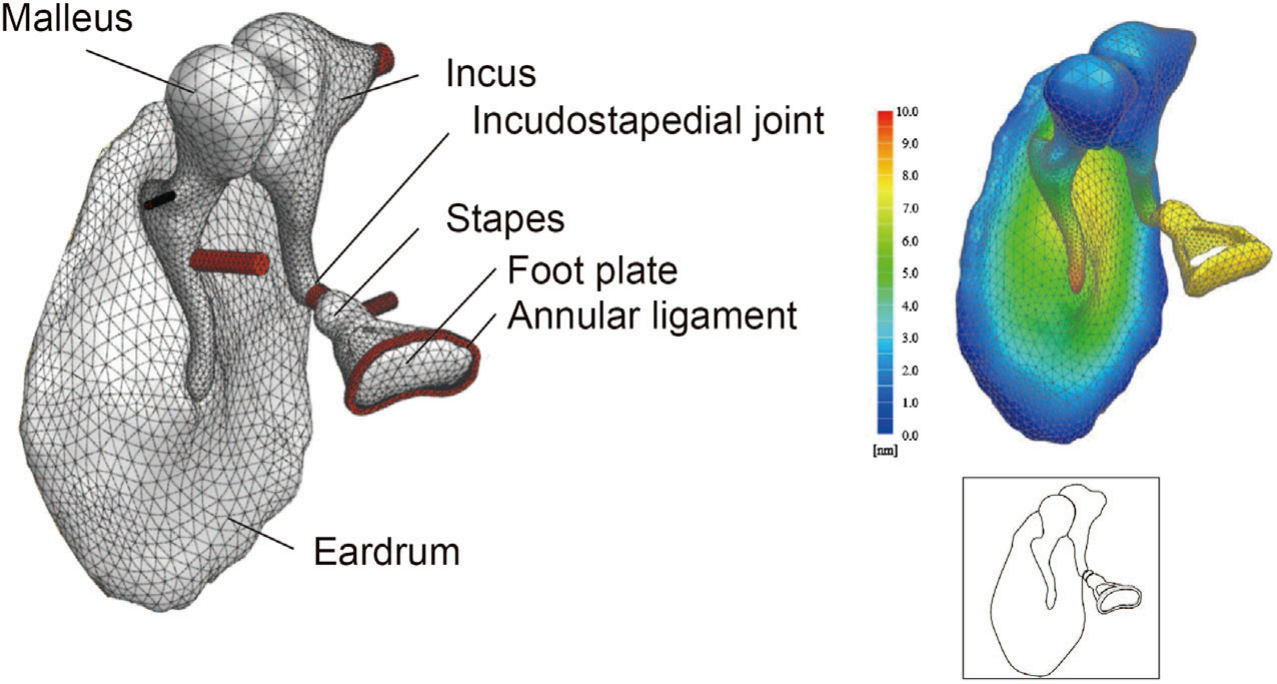
Finite element model (FEM) of the middle ear. Even complex shapes such as the eardrum and ear ossicles can be represented by a finite set of tetrahedron elements (49552 elements in this model). The displacement at any point can be measured by applying a set sound pressure, and the amount of displacement is displayed as a heat map. Illustrations such as that shown in the lower-right panel are used throughout this paper to summarize the FEM.

Morphological data for the tympanic membrane and ossicles were obtained from the micro-CT imaging study of Sieber et al. (Sieber et al., 2019). The tendons and ligaments attached to the ossicles were modeled as cylinders using dimensions measured by micro-CT imaging (Cheng and Gan, 2007; Sim and Puria, 2008). The tympanic membrane was modeled with spring elements set around it, and the tympanic membrane, ossicles, tendons, and ligaments (anterior ligament of the malleus, posterior ligament of the incus, tensor tympani muscle, annular ligament of the stapes and stapedius muscle) were set as elastic bodies with physical parameters of density and Young’s modulus. Since the footplate of the stapes pushes against the oval window, which is in contact with the perilymph of the cochlea, the model included a viscous damping coefficient (assuming a fluid damper). The settings and physical property values were selected with reference to the study of Koike et al. (Koike et al., 2002), but the parameters related to damping were modified (Supplementary Table S1). The equations were analyzed using the Simcenter Nastran general-purpose structural analysis program (Siemens, Germany). The equation used was:

$$\left(\left[\begin{array}{cc} K_{s} & -C\\ 0 & K_{A} \end{array}\right]+j\omega \left[\begin{array}{cc} D_{s} & 0\\ 0 & D_{A} \end{array}\right]-\omega^{2}\left[\begin{array}{cc} M_{s} & 0\\ C^{T} & M_{A} \end{array}\right]\right)\left\{\begin{array}{c} x\\ p \end{array}\right\}=\left\{\begin{array}{c} f_{s}\\ f_{A} \end{array}\right\}$$

where *K* is the stiffness matrix, *D* is the damping matrix, *M* is the mass matrix, *C* is the coupling matrix, *x* is the displacement vector, *p* is the sound pressure vector, *f* is the external force vector, _*S*_ denotes the structural component and _*A*_ denotes the acoustic component. The ear canal and middle ear cavity were not included in the FEM. The present FEM excluded the middle ear cavity because a review of the literature indicated that the magnitude of the impedance of the middle ear cavity is negligible compared to the magnitude of the impedance of the tympanic membrane and ossicles; this allowed us to concentrate on the mechanically most important structures in the middle ear (Zwislocki, 1962; Homma et al., 2009). The FEM of the middle ear is illustrated in Figure 1.

### Conversion of data generated by the finite element model into an audiogram

**FIGURE 2:**
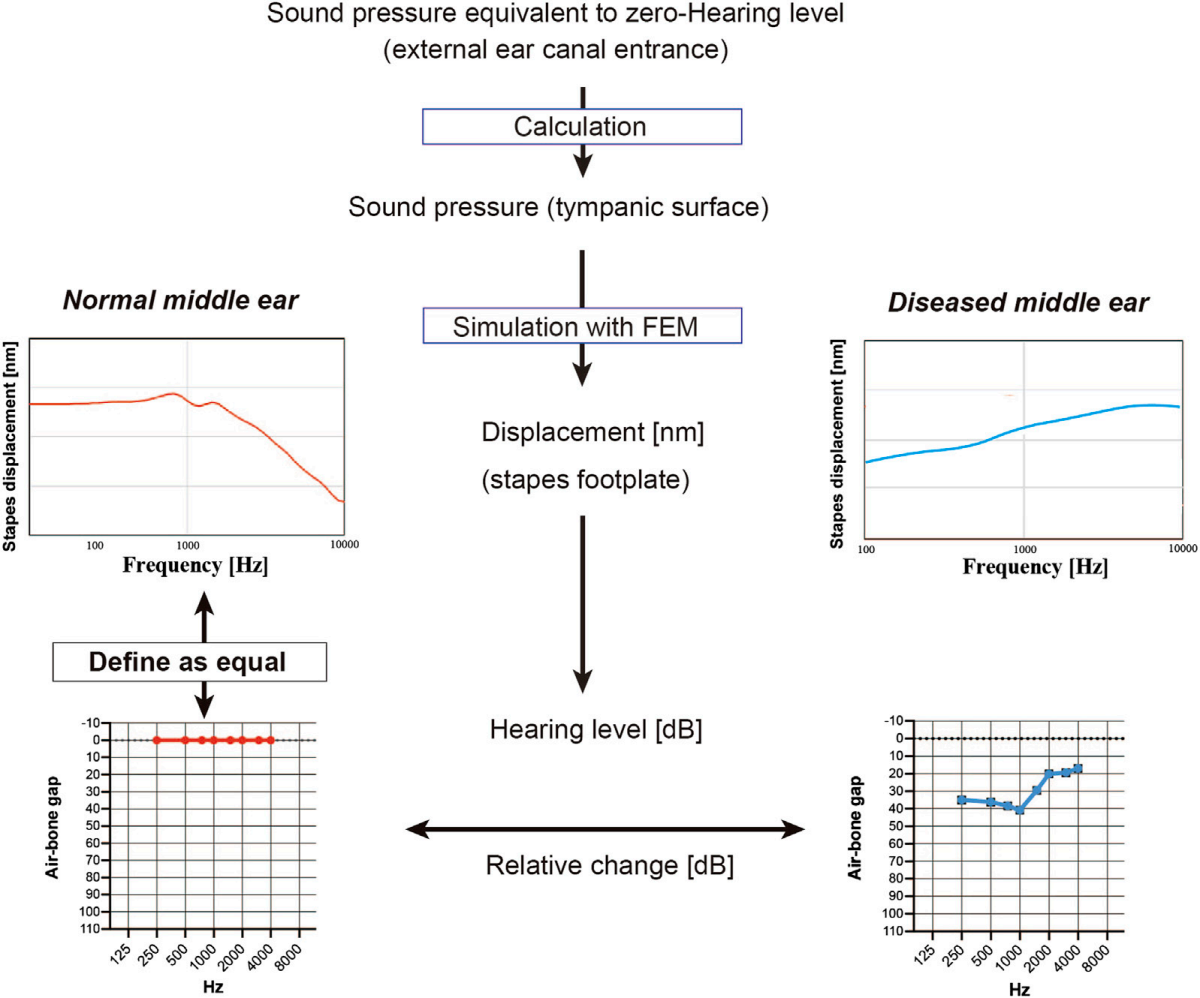
Simulation of the audiogram using data from the finite element model (FEM). To mimic a hearing test, the sound pressure on the tympanic membrane when the minimum audible sound pressure of each frequency was applied from the entrance of the ear canal was calculated, and the FEM of the normal middle ear was used to simulate the displacement of the stapes footplate at each frequency; these values were used to define the zero hearing level in the audiogram. Values for stapes footplate displacement calculated for the FEMs of diseased middle ears were expressed relative to those of the normal middle ear and were represented on the simulated audiogram as the air-bone gap.

This section describes the scheme used to convert the frequency characteristics of the stapes footplate displacement obtained from the FEM into an audiogram (Figure 2). First, the sound pressure at which the hearing level (HL) was zero was calculated from the reference equivalent threshold sound pressure level of the audiometer (AA-78, Rion, Japan). The gain obtained in the ear canal for each frequency (Ballachanda, 1997) was added to this and set over the tympanic surface of the middle ear. In the model of the normal middle ear, the pressure at the footplate of stapes was calculated at each frequency, and this was defined as a HL of 0 in the audiogram. The models of diseased middle ears calculated the HL in dB based on the displacement of the stapes bone relative to that of the model of the normal middle ear, which theoretically corresponds to the ABG on the audiogram. The frequencies used for the FEM included 250, 500, 1000, 2000, and 4000 Hz (the frequencies at which the ABG is measured in the clinical setting) as well as 750, 1500, and 3000 Hz.

### Clinical data and definition of audiometric pattern

The use of clinical data in this study was approved by The Jikei University ethics committee (reference number 32-205/10286). The study included patients with malformation of the ossicular chain, traumatic ossicular chain disruption or otosclerosis, confirmed mobility of the stapes footplate and normal tympanic findings who underwent middle ear surgery at the Jikei University School of Medicine between April 2016 and March 2020. The diagnosis was made on the basis of the medical history (congenital anomalies, head injury, etc.), otoscopy findings, pure-tone audiometry and CT imaging and was confirmed at the time of middle ear surgery. Pure-tone audiometry was performed in a double-chamber anechoic room. All hearing tests were performed by an experienced audiologist who measured air conduction and bone conduction thresholds with masking as appropriate. Hearing tests were performed preoperatively using frequencies of 250, 500, 1000, 2000, and 4000 Hz. An otolaryngologist confirmed the details of the thresholds. The number of cases and age distribution by disease are presented in Table 1. Audiometric sloping was defined as a difference in HL of more than 10 dB between the mean value for 250–500 Hz (a) and the mean value for 2000–4000 Hz (b), with the HL for 1000 Hz being between a and b (Farahmand et al., 2016).

**TABLE 1:** Data for the clinical cases included in the study.

Clinical diagnosis		Age, years (mean ± SD)	Number (male:female)	Air-bone gap, dB HL (mean ± SD)
Otosclerosis (raw data, no correction at 2000 Hz)		46.3 ± 5.9	24 (11:13)	28.3 ± 11.4
Fixation of malleus and/or incus (malformation)		18.3 ± 10.5	7 (6:1)	42.8 ± 11.2
Incus-stapes discontinuity	Trauma	36.0 ± 11.7	5 (4:1)	24.6 ± 9.7
	Malformation	29.8 ± 13.7	8 (3:5)	43.0 ± 9.74

## Results

### Validity of the finite element model

**FIGURE 3:**
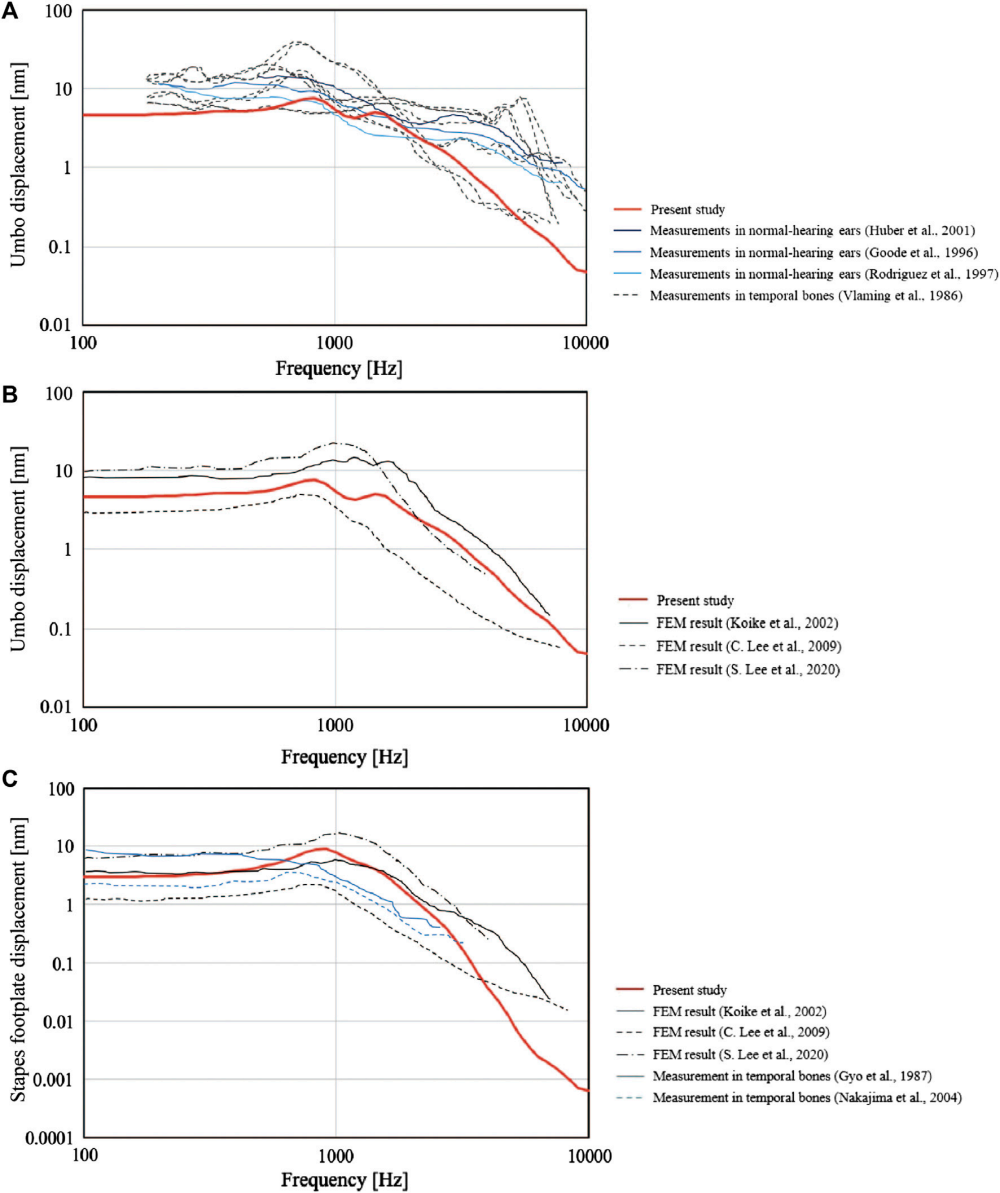
Comparisons between the present finite element model (FEM) and previously published data. The frequency-response characteristics of the tympanic membrane (umbo) and the stapes bone under 80 dB SPL sound pressure are shown. The values obtained by the FEM in the present study (solid red line) were similar to those reported previously. **(A)** Measured values for the perpendicular displacement of the tympanic membrane (umbo). **(B)** Simulated values for the perpendicular displacement of the tympanic membrane (umbo) calculated using FEMs. **(C)** Measured and simulated (FEM) values for the perpendicular displacement of the stapes footplate.

First, the validity of the newly constructed FEM was evaluated through comparisons with previously published data. To achieve this, we used the FEM to calculate the displacements of the malleus umbo and stapes footplate perpendicular to the structural plane during the simulation of an 80 dB SPL sound pressure applied to the tympanic surface. Then, the calculated values were compared with those measured previously in the living body or cadavers and with data published for other FEMs. We compared our simulation values for the measured displacement of the tympanic membrane (umbo) (Figure3A), the simulated values of the tympanic membrane (umbo) calculated using FEM (umbo) (Figure3B) and the measured values of the stapes foot plate (Figure3C). We found that the curves obtained using our FEM were similar to those described in the literature (Vlaming and Feenstra, 1986; Gyo et al., 1987; Goode et al., 1996; Rodriguez Jorge et al., 1997; Huber et al., 2001; Koike et al., 2002; Nakajima et al., 2004; Lee et al., 2006; Volandri et al., 2011; Lee, 2020).

### Comparisons of the air-bone gaps for sclerotic pathologies between finite element models and clinical cases

**FIGURE 4:**
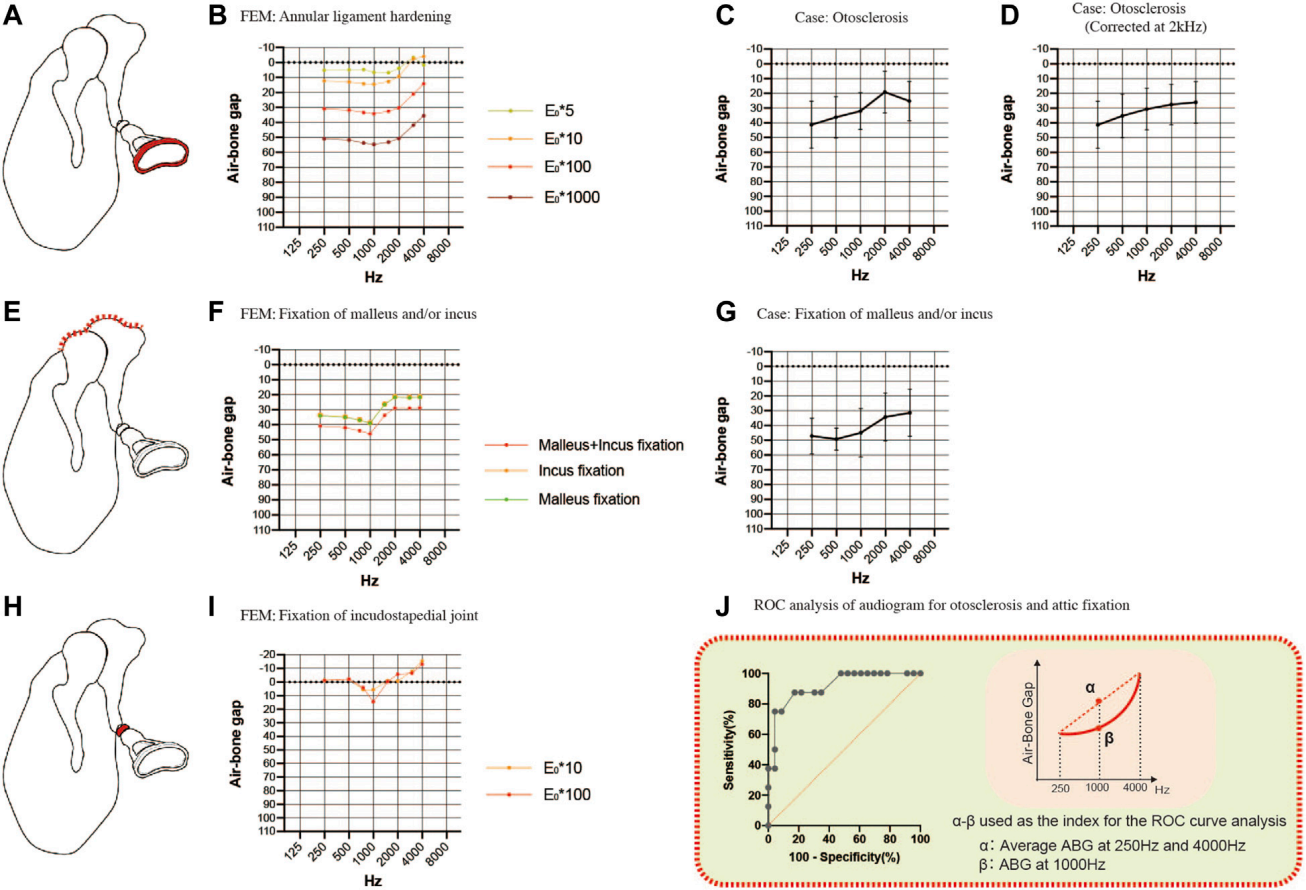
Comparisons of the air-bone gaps (ABGs) for sclerotic pathologies between the FEM and clinical cases. **(A)** Schematic showing annular ligament hardening (red). **(B)** Audiogram simulated using the FEM of annular ligament hardening. **(C)** Averaged audiogram obtained from 24 cases with otosclerosis. The mean ABG was 29.1 ± 10.9 dB HL (*n* = 24). **(D)** Smooth curve obtained when the audiogram in **(C)** was corrected for the 2 kHz bone conduction threshold. **(E)** Schematic showing fixation of the malleus and/or incus (red). **(F)** Audiogram simulated using the FEM of malleus and/or incus fixation. **(G)** Averaged audiogram obtained from 7 patients with fixation of the malleus and/or incus. The mean ABG was 42.9 ± 11.2 dB HL (*n* = 7). **(H)** Schematic showing fixation of the incudostapedial joint (red). **(I)** Audiogram simulated using the FEM of incudostapedial joint hardening. **(J)** Although both otosclerosis and attic fixation were associated with an up-sloping audiogram, these two pathologies could be differentiated from each other by calculating the difference between the ABG at 1000 Hz and the mean of the ABGs at 250 and 4000 Hz. Receiver operating characteristic (ROC) curve analysis determined that the optimum cut-off value for this parameter was 13.8 dB HL (area under the curve: 0.916; *p* = 0.0006). **(E)** Young’s modulus.

Next, we investigated sclerotic pathologies (Figure 4). FEA was carried out by increasing the Young’s modulus of the stapedial annular ligament 5-fold, 10-fold, 100-fold or 1000-fold (Figures 4A,4B) to simulate the pathology of otosclerosis. A 10-fold change in the Young’s modulus produced an ABG of about 10 dB HL between 250 and 2000 Hz with little effect at 4000 Hz. Further increases in Young’s modulus did not change the shape of the audiogram, and a 1000-fold increase in the Young’s modulus caused an ABG of about 50 dB HL in the 250–2000 Hz frequency band. Analysis of audiograms obtained from 24 patients with otosclerosis revealed an elevated bone conduction threshold at 2000 Hz, which is known as Carhart’s notch (Figure 4C). Graphing of the average ABG at each frequency for the clinical cases yielded a curve with an up-slope that was similar to that for the FEM (Figure 4D shows the smooth curve obtained after correction for the bone conduction threshold at 2000 Hz). The average ABG for the clinical cases was 29.1 ± 10.9 dB HL, which would be consistent with an increase in the Young’s modulus of about 100-fold according to the results obtained with the FEM.

To simulate malformations in which the malleus and/or incus are fixed to the attic wall of the middle ear, we constructed FEMs with part of the upper surface of the malleus and/or incus set to non-vibration (Figure 4E). The up-sloping audiogram simulated by this FEM was similar to that for the FEM of otosclerosis. The ABG was 35 dB HL at frequencies below 1000 Hz and 20 dB HL at frequencies above 2000 Hz when only one of the bones was fixed, and an additional 10 dB HL was observed when both bones were fixed (Figure 4F). By comparison, the mean audiogram of 7 clinical cases with attic fixation showed an ABG of 50 dB HL at frequencies below 1000 Hz and about 30 dB HL at frequencies above 2000 Hz (Figure 4G). The average ABG for the 7 cases with attic fixation was 42.9 ± 11.2 dB HL, which was greater than that for the cases with otosclerosis.

The common audiometric features of the above sclerosing conditions were up-sloping due to ABG expansion in the low-frequency band and a strong gradient change (i.e., a dip) at around 1000 Hz. The audiogram for footplate fixation (otosclerosis) exhibited an exponential slope after correcting for the Carhart notch by substituting the value at 2000 Hz for the average value of the bone conduction thresholds at 1000 and 4000 Hz, while the audiogram for attic fixation had a sigmoidal shape (Figures 4D,G; the averaged raw values for bone conduction thresholds used to correct the data for otosclerosis were 25.0 dB HL at 1000 Hz, 35.2 dB HL at 2000 Hz and 26.3 dB HL at 4000 Hz). By taking advantage of the distinctive features of these audiograms, receiver operating characteristic (ROC) curve analysis was able to distinguish attic fixation from footplate fixation (otosclerosis) based on whether the ABG at 1000 Hz was more than 13.8 dB (optimal cut-off value) above the mean of the ABGs at 250 and 4000 Hz (Figure 4J). Using this optimal cut-off value, the ROC curve analysis yielded an area under the curve value of 0.916 (*p* = 0.0006), a sensitivity of 87.5% and a specificity of 82.6%.

Incudostapedial joint sclerosis may be caused by manipulation of the joint (the incudostapedial joint is temporarily removed and repositioned in some tympanoplasty and facial nerve decompression procedures) or chronic inflammation. Although there were no corresponding clinical cases in the present study, we simulated the effects of sclerotic lesions of the incudostapedial joint by increasing the Young’s modulus 10-fold or 100-fold (Figures 4H,I). A characteristic dip appeared at a frequency of 1000 Hz irrespective of the Young’s modulus value used.

### Comparisons of the air-bone gaps for discontinuous pathologies between finite element models and clinical cases

**FIGURE 5:**
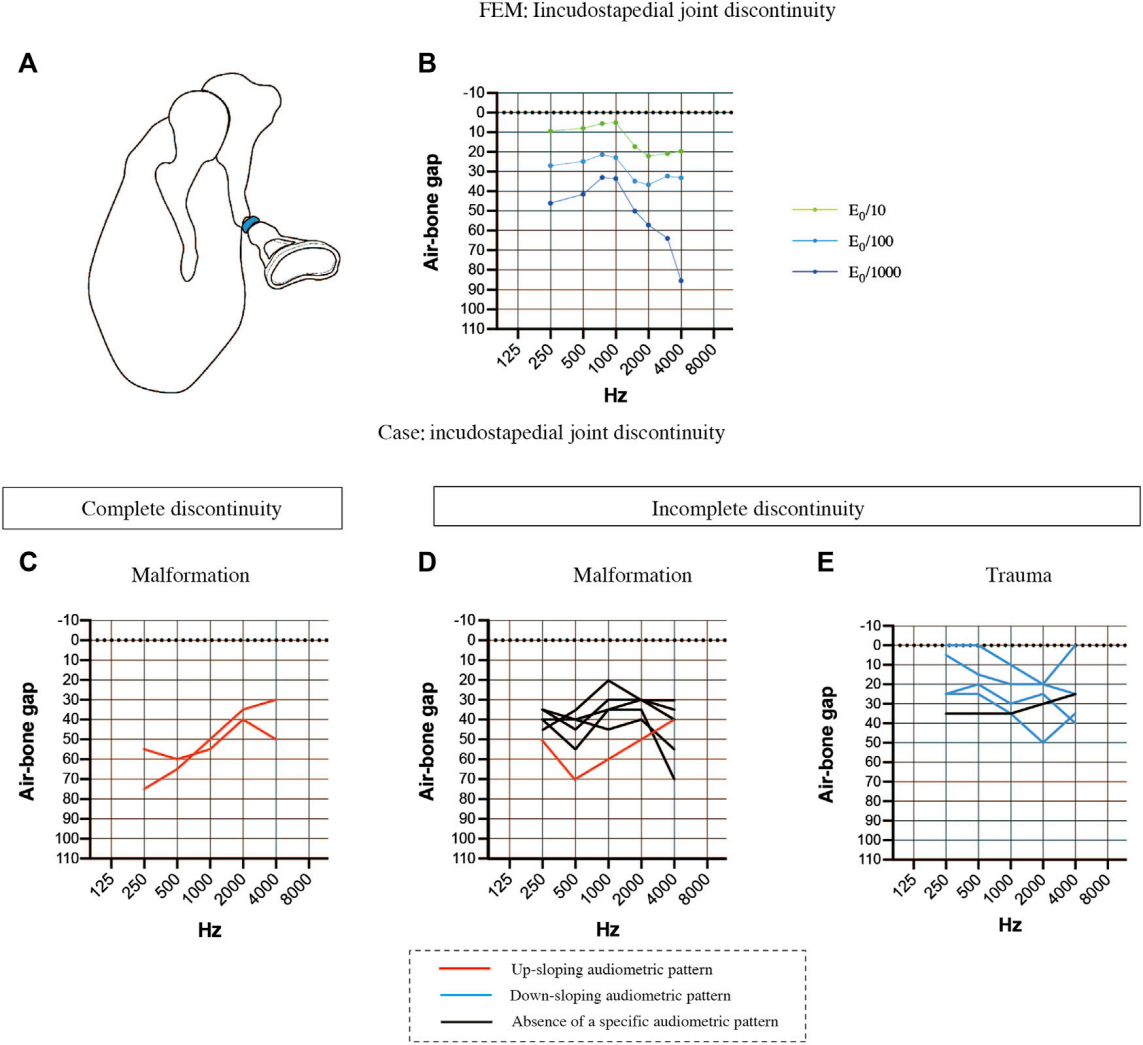
Comparisons of the air-bone gaps (ABGs) for discontinuous pathologies between the FEM and clinical cases. **(A)** Schematic showing complete discontinuity of the incudostapedial joint (blue). **(B)** Audiogram simulated using the FEM of incudostapedial joint instability. A 10-fold decrease in the Young’s modulus (E_0_) resulted in a down-sloping audiogram due to increases in the ABG at frequencies above 1000 Hz. A 1000-fold decrease in the Young’s modulus led to an even greater ABG at frequencies above 1000 Hz as well as an increase in the ABG at 250 and 500 Hz, leading to a prominent peak at 750–1000 Hz. **(C)** Averaged audiogram for clinical cases with complete discontinuity of the incudostapedial joint due to malformation. **(D)** Averaged audiogram for clinical cases with incomplete discontinuity of the incudostapedial joint due to malformation. **(E)** Averaged audiogram for clinical cases with incomplete discontinuity of the incudostapedial joint due to trauma. All patients with a down-sloping audiogram, shown as blue lines in **(E)**, had incomplete discontinuity of the incudostapedial joint due to trauma, and the mean ABG for these patients was 22.5 ± 5.7 dB HL (*n* = 4). The mean ABG for the patients with an up-sloping audiogram, shown as red lines in **(C)** and **(D)**, was 53.9 ± 9.6 dB HL (*n* = 3). The mean ABG for the patients without a sloping audiogram was 35.8 ± 4.1 dB HL (*n* = 6). A slope was defined as a difference of more than 10 dB between (a) the mean of the ABG values at 250 and 500 Hz and (b) the mean of the ABG values at 2000 and 4000 Hz, with the ABG value at 1000 Hz lying between (a) and (b).

Then, we examined the effects of ossicular chain discontinuity (Figure 5). FEMs of incudostapedial joint discontinuity were generated by decreasing the Young’s modulus 10-fold, 100-fold or 1000-fold (Figure 5A). The ABG was about 20 dB HL between 2000 and 4000 Hz when the Young’s modulus was reduced by 10-fold, resulting in a down-sloping simulated audiogram. The down-slope at frequencies above 1000 Hz was steepened when the Young’s modulus was further reduced, and the ABG at frequencies above 2000 Hz exceeded 60 dB HL. On the other hand, the ABG at frequencies between 250 and 750 Hz was less affected by reductions in the Young’s modulus and was about 10 dB HL for a 10-fold decrease in the Young’s modulus and around 40 dB HL for a 1000-fold decrease in the Young’s modulus. Furthermore, the curve between 250 and 750 Hz was up-sloping in contrast to the down-slope seen at frequencies above 1000 Hz. As a result, the peak at 750–1000 Hz became more prominent as the Young’s modulus was decreased.

The audiological findings were highly variable in 13 patients with suspected incudostapedial discontinuity and an intraoperative diagnosis. The patients were divided into the following groups based on the type of incudostapedial joint discontinuity (complete or incomplete) and the pathogenesis: complete discontinuity of the incudostapedial joint due to middle ear malformation (*n* = 2; Figure 5C), incomplete discontinuity due to middle ear malformation (*n* = 6; Figure 5D) and incomplete discontinuity due to trauma (*n* = 5; Figure 5E). Cases with a down-sloping audiogram (Figure 5E, blue lines) had a mean ABG of 22.5 ± 5.7 dB HL (*n* = 4), and all had traumatic causes of incomplete discontinuity, with two cases described in the surgical records as having soft tissue bands at the point of discontinuity. Cases with an up-sloping audiogram (Figures 5C,D, red lines) had a mean ABG of 53.9 ± 9.6 dB HL (*n* = 3), and all three cases had malformations. Two of these cases had complete discontinuity of the ossicular chain, one due to sclerosis of the incudostapedial joint and the other due to discontinuity of the posterior limb of stapes. Cases without a clear up-sloping or down-sloping audiogram (Figure 5D, black lines) had an intermediate mean ABG of 35.8 ± 4.1 dB HL (*n* = 6). The majority of these cases (*n* = 5/6) were malformations, and all were described as having soft tissue at the point of discontinuity. Three of the six non-sloping audiograms exhibited a shoulder at 1000 Hz (ABG at least 10 dB HL lower than the average of the ABGs at the adjacent frequencies). Thus, it is possible that a combination of factors may be responsible for the impairment of sound transmission in patients with middle ear malformations whose audiograms do not show a clear up-slope or down-slope.

### Simulated air-bone gaps for finite element models of changes in ossicular mass

**FIGURE 6:**
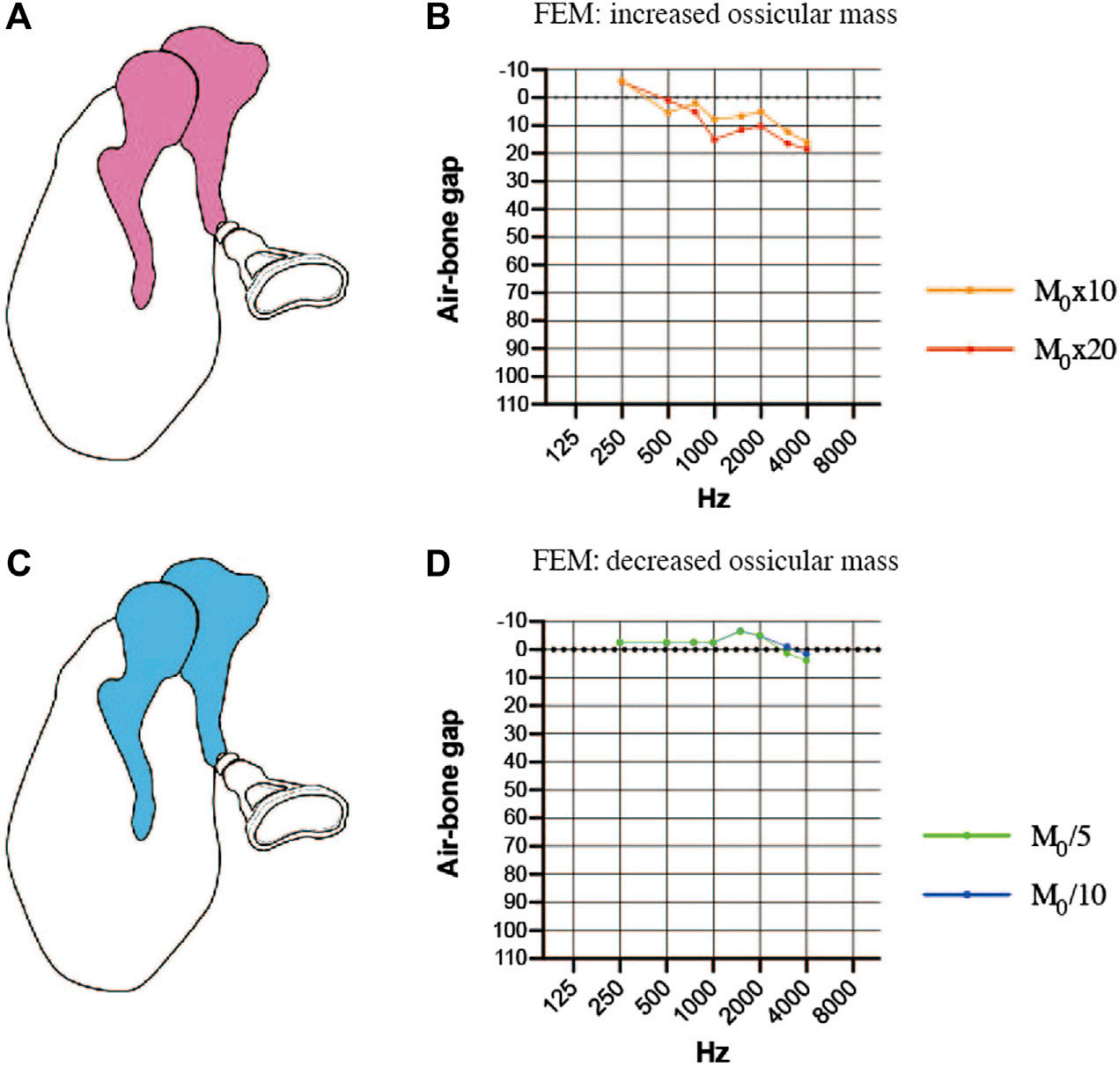
Simulated air-bone gaps (ABGs) for finite element models (FEMs) of altered malleus and incus mass. **(A)** Schematic showing an increase in the mass of the malleus and incus (pink). **(B)** Audiogram simulated using the FEM of increased ossicular mass. **(C)** Schematic showing a decrease in the mass of the malleus and incus (blue). **(D)** Audiogram simulated using the FEM of decreased ossicular mass. The effects of the mass change were mainly observed in the 1000–4000 Hz frequency range, but the changes in the ABG were less than 10 dB HL for a 10-fold increase or decrease in mass. M_0_: mass.

We also explored whether changes in ossicular mass (a 10-fold or 20-fold increase or a 5-fold or 10-fold decrease) would affect the efficiency of sound transmission. Increasing the mass of the incus and malleus in the FEM (Figure 6A) resulted in an increase in the ABG at frequencies between 1000 and 4000 Hz and a down-sloping simulated audiogram (Figure 6B). The ABG between 1000 and 4000 Hz was about 10 dB HL for a 10-fold increase in mass and around 15 dB HL for a 20-fold increase in mass. The ABG between 1000 and 4000 Hz was reduced when the mass of the incus and malleus was decreased (Figure 6C), but the effect was less than 10 dB HL even when the mass was reduced 10-fold, and there was no sloping tendency in the curve (Figure 6D).

### Simulated air-bone gaps for finite element models of complex pathologies

**FIGURE 7:**
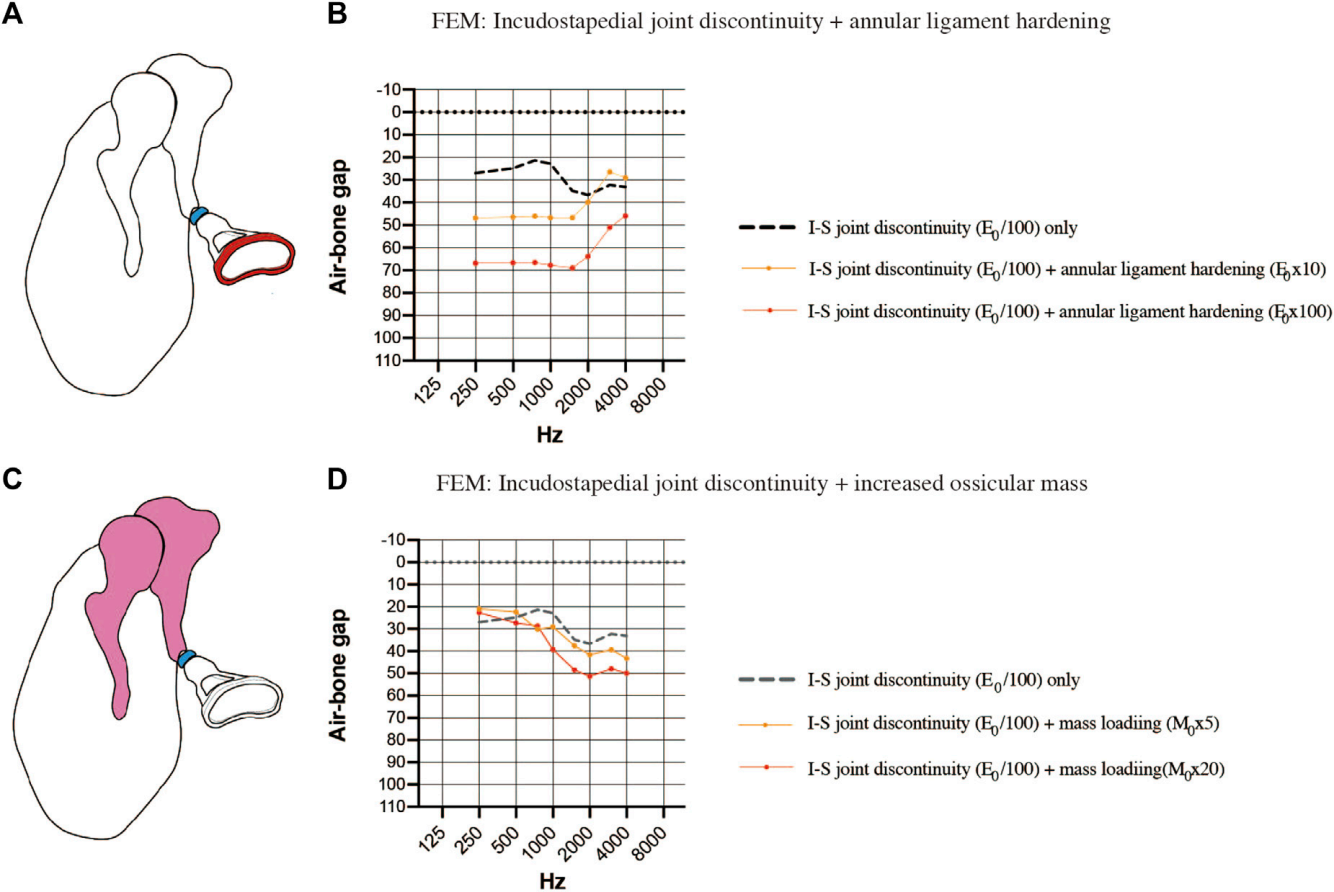
Simulated air-bone gaps (ABGs) for complex pathologies. **(A)** Schematic showing the combination of incudostapedial joint discontinuity and sclerosis of the stapes footplate. **(B)** Audiogram simulated using the FEM showing the effects of stapes footplate sclerosis (Young’s modulus of the annular ligament increased 10-fold or 100-fold; red) superimposed on incudostapedial joint discontinuity (Young’s modulus decreased 100-fold; blue). There was an additional increase in the ABG at frequencies of 250–1500 Hz when the stiffness of the annular ligament was increased. **(C)** Schematic showing the combination of incudostapedial joint discontinuity and increased mass of the incus and malleus. **(D)** Audiogram simulated using the FEM showing the effects of ossicular mass increase (mass of the incus and malleus increased 5-fold or 20-fold; pink) superimposed on incudostapedial joint discontinuity (Young’s modulus decreased 100-fold; blue). The ABG was increased when ossicular mass was elevated, but the basic features of the audiogram pattern remained. E_0_: Young’s modulus; I-S: incudostapedial joint; M_0_: mass.

Finally, the coexistence of multiple pathologies was considered. The first FEM evaluated the combination of incudostapedial joint discontinuity and sclerosis of the stapes footplate (Figure 7A). When sclerosis of the stapes footplate (Young’s modulus of the annular ligament increased 10-fold or 100-fold) was added to the FEM of incudostapedial joint discontinuity (Young’s modulus reduced 100-fold), there was an additional increase in the ABG at frequencies of 250–1500 Hz of up to 40 dB HL (Figure 7B). The second FEM evaluated the combination of incudostapedial joint discontinuity and increased ossicular mass (Figure 7C). When a 5-fold or 20-fold increase in ossicular mass was added to the FEM of incudostapedial joint discontinuity, the ABG at frequencies of 1000–4000 Hz increased by an additional 10–15 dB HL (Figure 7D). The simulated audiogram was similar to the non-sloping audiogram observed in some of the cases of ossicular malformation described above. In both the FEMs that considered multiple pathologies, the general result was that the effects of one pathology on ABG were added to those of the other pathology.

## Discussion

In this study, we first constructed a FEM of the normal middle ear and validated it against findings published in the literature. Then, we used our FEM to explore the relationship between specific pathologies (including otosclerosis, ossicular fixation, ossicular chain discontinuity, ossicular mass change and combination pathologies) and the audiogram, and the results were compared to audiometric data from clinical cases where possible. Our findings demonstrate that the simulated audiometric pattern for each of the diseases modeled by our FEM was broadly consistent with the averaged audiogram obtained from clinical cases. We anticipate that the results of this study will help to develop analytical methods for pure-tone audiometry that will facilitate preoperative estimation of the pathology underlying conductive hearing loss.

The validity of the FEM was first assessed through comparisons with previously published data. The results obtained for the present FEM were similar to those reported previously for simulated tympanic membrane displacement (Figure 3B) and measured stapes footplate displacement (Figure 3C). However, our results at the very highest and very lowest frequencies showed some variation from previous measurements of tympanic membrane displacement (Figure 3A). A possible reason for the small inconsistencies at the highest and lowest frequencies is that the tympanic membrane is prone to variations in its physical properties and hence is likely to show inter-individual differences. Nevertheless, since the present study was focused on trends in the slope of the audiogram, and since the variations from the published data in Figure 3A mainly appeared at frequencies not considered in the present study (i.e., below 250 Hz and above 4000 Hz), we do not believe that these differences affect the main findings and conclusions of this study.

The sclerotic lesions of the ossicles analyzed in this study included fixation of the stapes footplate, fixation of the malleus and incus to the attic wall, and incudostapedial joint adhesion. The simulated audiograms obtained after FEA indicated that all these pathologies were associated with an up-sloping audiogram, and the results were consistent with audiometric data obtained from 31 clinical cases. Additionally, the FEA showed that the shape of the sloping audiogram differed between stapes footplate fixation and attic fixation, and the ABG at 1000 Hz helped to differentiate between these disorders.

Since complete discontinuity of the ossicular chain could not be simulated by the present FEM, the analysis focused on incomplete discontinuity of the incudostapedial joint. The results showed that the simulated audiogram exhibited a down-sloping ABG and a threshold that increased with the degree of discontinuity. Evaluation of 19 clinical cases with incudostapedial joint discontinuity revealed a variety of audiometric patterns. Patients with incomplete incudostapedial joint discontinuity due to trauma tended to have a down-sloping audiogram (*n* = 4/5) as predicted by the FEM, those with incomplete incudostapedial joint discontinuity due to middle ear malformation tended to have a moderate ABG (*n* = 5/8) without obvious sloping of the audiogram, and those with complete incudostapedial joint discontinuity due to middle ear malformation had an up-sloping audiogram with an ABG of about 60 dB HL at low frequencies and around 40 dB HL at high frequencies (*n* = 2/2). We consider incomplete discontinuity of the incudostapedial joint due to trauma to represent a single lesion, whereas the ABG changes associated with middle ear malformations may result from multiple lesions. The up-sloping ABG was similar between cases of complete incudostapedial joint discontinuity, and we consider the changes in ABG to result from a mechanism that did not directly involve the ossicles. In addition, we were able to model the effects of changes in ossicular mass and simulate the audiograms for overlapping sclerotic and discontinuous lesions. Schematic representations of the audiograms for each of these conditions are shown in Figures 5C–E–5E.

The ABG reflects the difference between air-conduction hearing tests and bone conduction hearing tests and thus can be used to assess the severity of hearing loss due to middle ear pathology. However, an important finding of the present study is that the various pathologies differed in terms of their effects on the shape of the ABG, including features such as sloping, peaks and dips. This raises the possibility that these features of the ABG potentially could be used to facilitate the diagnosis of middle ear pathology.

**FIGURE 8:**
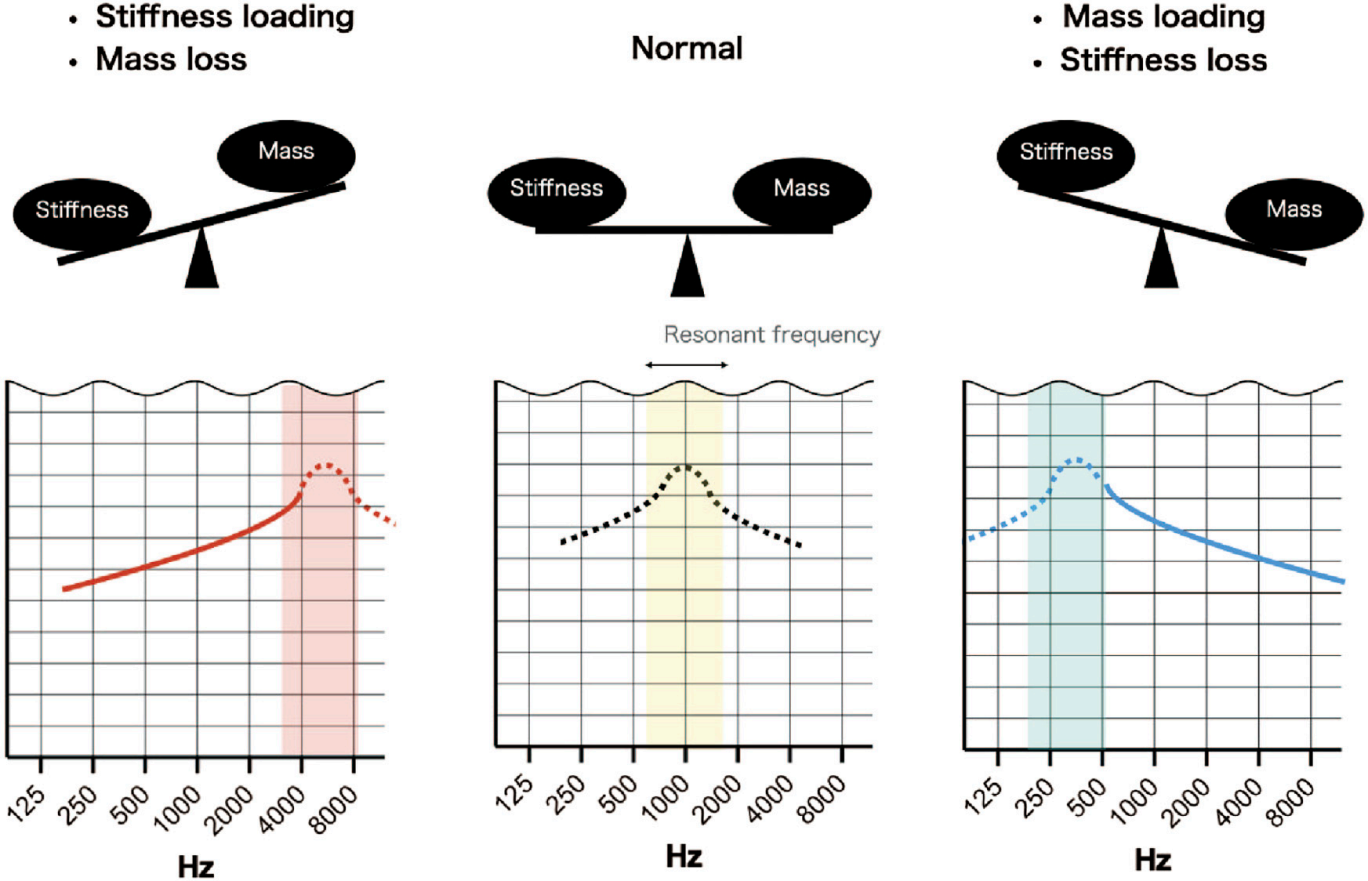
Simplified scheme illustrating the effects of stiffness and mass on resonant frequency. Normally, the resonant frequency is around 1000 Hz, but if sound transmission by the middle ear is normal, it is not detected due to calibration of the audiometer. An increase in stiffness and a loss of mass will shift the resonant frequency to a higher frequency and result in an up-sloping audiogram. A decrease in stiffness and a gain in mass will shift the resonant frequency to a lower frequency and result in a down-sloping audiogram.

Understanding why certain pathologies cause sloping of the audiogram requires consideration of the resonant frequency of the middle ear. The resonant frequency of the middle ear can be estimated by wideband absorptiometry (WBA) and is approximately 1000 Hz (800–1200 Hz) in normal subjects (Silman and Silverman, 1991; Wada et al., 1998). The audiogram used in clinical practice is calibrated to display as a flat line when the hearing level is normal so that hearing loss is easily recognized. Therefore, when the bone conduction threshold is normal, the audiogram has no slope. A sloping audiogram is caused by a change in acoustic impedance (due to pathology) that shifts the resonant frequency to a higher or lower level. For example, the resonant frequency shifts to a lower frequency (around 750 Hz) with ossicular dehiscence and to a higher frequency (around 1400 Hz) with otosclerosis (Karuppannan and Barman, 2021). The main factors influencing impedance and resonant frequency are stiffness, which affects low frequencies, and mass, which affects high frequencies (Kim and Koo, 2015). A seesaw analogy has been used to describe the balance between stiffness and mass in terms of their effects on the shape of the audiogram (Johansen, 1948) (Figure 8). In addition to sloping, peaks and dips were observed at certain frequencies in some of the simulated audiograms and some of the audiograms obtained from patients. Peaks and dips in the audiogram are generally not of great clinical interest, but their presence in an audiogram and the frequency at which they appear can provide important information about the resonant frequency. Since the calibration value is different for each frequency, a shift in the resonant frequency can result in a calibrated curve, that is, no longer smooth and contains a peak or a dip at a certain frequency. Therefore, the evaluation of peaks, dips and slopes can provide useful information that may help to distinguish between middle ear pathologies. Below, we discuss the possible pathologies underlying the different slope types in the audiogram.

### Up-sloping audiometric pattern

An up-sloping audiometric pattern results from an increase in the stiffness component or a decrease in the mass component. Clinically, otosclerosis (fixation of the footplate of stapes) is known to cause an up-sloping audiogram, and an up-slope would also be expected if the stiffness component increased due to attic fixation of the malleus and incus (Schlauch and Nelson, 2014). However, analysis of the FEMs revealed that the features of the up-sloping audiogram differed between fixation of the stapes footplate (otosclerosis) and attic fixation of the malleus and incus. Specifically, the audiogram exhibited an exponential slope in the 1000–2000 Hz frequency range for stapes footplate fixation but a sigmoidal slope for malleus or/and incus fixation. Furthermore, ROC curve analysis identified an optimal cut-off value for a parameter (difference between ABG at 1000 Hz and the mean of the ABGs at 250 and 4000 Hz) that could be used to help distinguish these pathologies from each other. Additional research with a larger number of cases may allow the determination of a more accurate cut-off value.

Cases with complete discontinuity of the incudostapedial joint also had an audiogram with a large up-slope, but the pathology could not be analyzed with our FEM because our simulation required the transmission of mechanical vibrations between the ossicles. The two cases with complete discontinuity had an ABG above 60 dB HL at 500 Hz and very similar audiogram slopes, which may reflect the bone conduction of sound transmitted through the headphones during the air conductivity measurement and the limit of the ABG that could be measured with a standard pure-tone audiometer. Although a conductive threshold is involved in the ABG, there is some debate regarding the factors that underlie its mechanics. Farahmand et al. (2016) reported an up-sloping ABG for a case with complete ossicular dissection. In addition, these authors used an artificial material (Jeltrate) to firm up the discontinuity of the cadaveric ossicle and attempted to compare complete and incomplete discontinuity through Doppler measurements of stapes vibration. Their results showed that incomplete discontinuity was associated with dampening of only high frequencies and a relatively low average ABG, whereas complete discontinuity was also associated with heavy dampening of low frequencies. Our FEM of incomplete discontinuity indicated that the ABG at 500 Hz was around 40 dB HL even when the stiffness of the incudostapedial joint was reduced 1000-fold, so in clinical practice it would be reasonable to suspect complete discontinuity if the ABG reached 60 dB HL at low frequencies.

The clinical effects of a reduction in ossicular mass, which can arise due to deformities of the ossicles, are not often discussed. The results obtained with our FEM suggest that, if the continuity and rigidity of the ear ossicles are maintained, a decrease in ossicular mass improves the transmission of high-frequency vibrations and leads to an up-sloping audiogram, although the overall impact of lower ossicular mass on the slope of the audiogram is small.

### Down-sloping audiometric pattern

Analysis using the FEM showed that a down-sloping audiogram predominated when the stiffness of the incudostapedial joint was reduced and when the mass of the ossicles was increased. It has been reported previously that a down-sloping audiogram is a sign of incomplete discontinuity (Sim et al., 2013; Sarmento et al., 2017). The case of traumatic incudostapedial joint disruption described in the present study (Figure 5E) is also considered to be an incomplete discontinuity with a small ABG. Interestingly, when the degree of incudostapedial joint discontinuity was increased in the FEM, the down-slope of the audiogram was enhanced and the low-frequency threshold was increased, resulting in the formation of a peak between 750 and 1000 Hz. WBA measurements suggest that the resonant frequency is around 500–800 Hz in cases of ossicular disarticulation (Nakajima et al., 2012; Karuppannan and Barman, 2021), which would be consistent with the peak in our simulation and suggestive of a shift in the resonant frequency. Although 750 Hz is not a frequency, that is, usually evaluated in clinical practice, measurement of this frequency in cases of suspected incudostapedial discontinuity might provide valuable information.

The audiograms of patients with incudostapedial joint discontinuity showed wide variation, necessitating separate analyses of each disease type. Traumatic discontinuity presented with a down-sloping audiogram in most cases and, according to the FEM, could be treated as a single condition of incomplete incudostapedial discontinuity in many instances. On the other hand, all cases of middle ear malformation were associated with a non-down-sloping hearing pattern even when incomplete incudostapedial joint discontinuity was present. The audiograms of patients with middle ear malformations generally had a higher ABG than those of traumatic cases with incomplete incudostapedial joint discontinuity, and the reduced efficiency of sound transmission was likely due to a combination of factors.

Published reports indicate that malformations have an average ABG of about 40 dB HL (Thomeer et al., 2011; Vincent et al., 2016; Zhang et al., 2020), but traumatic incomplete incudostapedial discontinuity has an average ABG of around 20 dB HL (Sim et al., 2013). In addition, the ABG varies depending on the complexity of the malformation (Thomeer et al., 2011), and complex malformations are easily missed (Teunissen and Cremers, 1993) and have worse postoperative outcomes than single malformations (Quesnel et al., 2015).

On the other hand, an increase in the mass component can also result in a down-slope, but clinically this could be due to the attachment of granulation tissue, the presence of an effusion due to inflammation or the attachment of a cholesteatoma matrix. In the FEM, the mass of the ossicles needed to be increased by more than 10-fold to obtain a change in the ABG that exceeded 10 dB HL, which would correspond to a mass of more than 400 mg. Although it is not possible to make a direct comparison, we consider the effects of mass changes on hearing to be less than those of stiffness changes.

### Absence of a specific audiometric pattern

Since middle ear malformations are assumed to be complex pathologies, we used the FEM to evaluate the effects of combination pathologies on the simulated audiogram. Simultaneous modeling of incudostapedial dissection and footplate fixation showed that the ABGs of each pathology were superimposed and that the audiometric pattern was similar to that seen in patients with middle ear malformations. This suggests that evaluation of the audiometric pattern combined with CT-based assessments of ossicular morphology and attic fixation may allow preoperative estimation of the pathology through the use of a FEM.

### Pathologies producing a dip at 1000 Hz

Although there is no known disease in which only the incudostapedial joint hardens, it is conceivable that the stiffness of this joint increases when it is removed and repositioned during surgery. A dip occurred at 1000 Hz in the simulated audiogram when the incudostapedial joint was stiffened (Figure 4I) or when the masses of the incus and malleus were increased (Figure 6B). The common feature of all these pathologies is that the incus and adjacent malleus are subjected to a strong local restriction of vibration, suggesting that the large mass of the incus and malleus contributes significantly to the amplitude at around 1000 Hz, the resonant frequency of the normal middle ear.

### Further development of the finite element model

In this study, we have used FEM to simulate the audiograms for otosclerosis, middle ear malformation and traumatic injury, which are all causes of conductive hearing loss. The results are important because they demonstrate that specific pathologies are associated with characteristic audiogram features. The present study did not consider misalignment such as ossicular chain dislocation, hence the development of a FEM to evaluate this pathology remains a challenge for the future. Comprehensive modeling of different conditions with varying severities and simulation of the corresponding audiograms would provide further useful insights. Moreover, this information potentially could be used to develop a system that, in future, would help to predict the site and type of a middle ear lesion based on an individual patient’s audiogram. This method might be particularly effective in cases with complex pathologies, although the FEM will need to be modified to take into account additional factors such as tympanic membrane integrity and middle ear effusion.

As many cases of conductive hearing loss are not thought to be due to one simple defect (equivalent to a change in only one parameter in our FEM), it will be important to accumulate more cases to improve the accuracy of our model. Although we found notable correlations between the audiograms simulated by the FEM and those of actual clinical cases, the inclusion of more cases might help us to build a model that would estimate the underlying pathology more accurately. In addition, the diagnostic accuracy of the FEM might be improved by incorporating data from other auditory tests or imaging techniques and by focusing on the effects of multiple pathologies.

## Data Availability

The raw data supporting the conclusion of this article will be made available by the authors, without undue reservation.
