# Supplementary material for: Combined analysis of finite element model and audiometry provides insights into the pathogenesis of conductive hearing loss
Source: Front Bioeng Biotechnol. 2022 Sep 2;10:967475. doi: 10.3389/fbioe.2022.967475 (PMC9479494; doi:10.3389/fbioe.2022.967475)
Supplement: Supplementary file 1 [file Table1.DOCX]

Supplementary Material

**Supplementary Table 1. Values used for the physical properties in the finite element model.**

| **Young’s modulus** | |  | **Value** | **Unit** |
| --- | --- | --- | --- | --- |
|  | Pars tensa |  | 3.34×10^7^ | N/m^2^ |
|  | Pars flaccida | | 1.11×10^7^ | N/m^2^ |
|  | Tympanic membrane (both ends of malleus) | | 3.34×10^7^ | N/m^2^ |
|  | Tympanic membrane (center of malleus) | | 3.34×10^4^ | N/m^2^ |
|  | Anterior ligament of the malleus | | 2.1×10^7^ | N/m^2^ |
|  | Posterior ligament of the incus | | 6.5×10^5^ | N/m^2^ |
|  | Tensor tympani muscle | | 2.6×10^6^ | N/m^2^ |
|  | Annular ligament of the stapes | | 6/5×10^4^ | N/m^2^ |
|  | Stapedius muscle | | 5/2×10^5^ | N/m^2^ |
|  | Incudostapedial joint | | 6×10^5^ | N/m^2^ |
|  | Ossicles |  | 1.2×10^10^ | N/m^2^ |
| **Spring constants** | |  |  |  |
|  | Linear spring (upper tympanic membrane) | | 3×10^3^ | N/m |
|  | Linear spring (lower tympanic membrane) | | 1.5×10^5^ | N/m |
|  | Rotational spring (upper tympanic membrane) | | 3×10^-5^ | Nm/m |
|  | Rotational spring (lower tympanic membrane) | | 1×10^-4^ | Nm/m |
| **Structural damping coefficient** | | |  |  |
|  | Ossicles |  | 0.01 |  |
|  | Others |  | 0.5 |  |
| **Viscous damping constant for the cochlea** | | | 6.24×10^-1^ | Ns/m |
